# Host Factors in the Natural History of Chronic Hepatitis B: Role of Genetic Determinants

**DOI:** 10.1155/2022/6046677

**Published:** 2022-08-23

**Authors:** Turyadi, Billy Witanto, Korri E. El-Khobar, M. Luthfi Parewangi, M. Rezki Rasyak, Dhita P. Wibowo, Meta D. Thedja, Irawan Yusuf, Muh. Nasrum Massi, Ilhamjaya Patellongi, Din Syafruddin, David H. Muljono

**Affiliations:** ^1^Post Graduate School, Faculty of Medicine, Universitas Hasanuddin, Makassar, Indonesia; ^2^Eijkman Center for Molecular Biology, National Research and Innovation Agency, Jakarta, Indonesia; ^3^Faculty of Medicine, Universitas Hasanuddin, Makassar, Indonesia; ^4^Faculty of Medicine and Health, University of Sydney, Sydney, Australia

## Abstract

**Background:**

The host immune system plays an important role in hepatitis B virus (HBV) infection manifestation. Genetic polymorphisms of several inflammatory cytokines, including TNF-*α* and IL-10, have been associated with chronic hepatitis B (CHB) progression, although with contradicting results. CHB progression can be categorized into four phases, immune tolerance (IT), immune clearance (IC), low/no replicative (LR), and e-negative hepatitis (ENH), with HBeAg seroconversion as an important milestone. Here, we determined the association of TNF-*α* (rs1800629) and IL-10 (rs1800896 and rs1800872) SNPs in the context of CHB natural history progression, particularly to HBeAg seroconversion, in Indonesian CHB patients.

**Methods:**

A total of 287 subjects were recruited and categorized into distinct CHB phases based on HBeAg, viral load, and ALT levels. TNF-*α* and IL-10 SNPs were determined using PCR-RFLP and confirmed with direct sequencing. The association between SNP genotypes with CHB dynamics was determined using logistic regression presented as odds ratio (OR) with 95% CI.

**Results:**

No significant association was found between IL-10 -592A/C polymorphism and progression of IT and IC to LR, IT and IC to ENH, and LR to ENH phases in all the gene models. IL-10 rs1800896 and TNF-*α* rs1800629 could not be analyzed using logistic regression. Subjects' age (≥40 years old) was significantly associated with IT and IC to LR (OR: 2.191, 95% CI 1.067–4.578, *P* = 0.034), IT and IC to ENH (OR: 7.460, 95% CI 3.316–18.310, *P* < 0.001), and LR to ENH (OR: 5.252, 95% CI 2.010–14.858, *P* = 0.001). Male gender was associated with LR to ENH (OR: 4.077, 95% CI 1.605–11.023, *P* = 0.004).

**Conclusions:**

Age and male gender were associated with CHB phase progression instead of the TNF-*α* and IL-10 polymorphisms. It would be beneficial to study not only the effect of host determinants but also the viral factor to understand the mechanisms of CHB phase progression.

## 1. Introduction

Hepatitis B virus (HBV) infection is still a major global health burden. An estimate of 296 million people globally were living with chronic HBV infection, with 1.5 million new infections annually [1]. HBV infection causes a diverse range of disease manifestations from acute asymptomatic and chronic hepatitis B (CHB) to end-stage liver diseases including cirrhosis and liver cancer [2]. The natural history of HBV infection resulted from the interaction between the viral, host, and environmental factors. Based on these interactions, CHB natural history can be categorized into four phases, immune tolerance (IT), immune clearance (IC), low/no replicative (LR), and e-negative hepatitis (ENH). However, not all CHB patients will experience these phases, nor will they occur sequentially [3]. HBeAg seroconversion is an important milestone in CHB natural history, associated with viral clearance and recovery and reduced liver failure risk [4]. In addition, spontaneous HBeAg seroconversion occurring at a younger age in perinatally infected HBV patients is associated with low rate of cirrhosis progression [5]. However, recent studies have shown that HBeAg loss in CHB patients may be associated instead with worsening of disease progression [6]. The proportion of these HBeAg-negative CHB patients also keep on increasing in the last two decades [3, 7].

HBV disease manifestation is also greatly affected by the age of the initial infection, where perinatal infection mostly resulted in long-life persistence and chronicity, while infection at adulthood is mostly self-limited [8]. A high rate of perinatal HBV transmission usually occurred in HBV endemic countries, including Indonesia, which has around 7.1% national rate of HBsAg positivity [9].

Liver damage during CHB occurred mostly through the host's immunological processes inducing chronic inflammatory events [10]. Thus, a specifically tailored immune regulation, through control of cytokine expression, is required to mount an effective viral clearance response without exacerbating the liver inflammation [10, 11]. Several cytokines have been associated with viral clearance and chronic HBV infection including IFN-*γ*, TGF-*β*, TNF-*α*, IL-10, IL-18, and IL-28B [2, 12, 13]. Host genetic polymorphisms of these genes have been shown as an important factor in determining the susceptibility to and disease outcome of HBV infection in different populations [2, 12, 14–19], albeit with conflicting results.

TNF-*α*, a proinflammatory cytokine, is greatly involved in the pathophysiology of viral hepatitis as mediator of a specific CD8+ T cells [2, 12]. TNF-*α* circulating level increased during HBV infection [20] and associated with inhibition of HBV replication in a transgenic mouse model most likely through the NF-*κβ* pathway [21]. The G>A polymorphism at position -308 of TNF-*α* promoter was associated with higher TNF-*α* serum level in acute HBV infection in Iranian population [18]. However, SNP TNF-*α* -308 heterozygote and minor genotypes (AA) were strongly associated with HBV infection resolution in Korean population [22], while G allele was associated with HBV persistence [23].

IL-10, an anti-inflammatory cytokine, regulates CD4+ T cell proliferation [13, 24], which are important for viral-specific control of HBV infection [11]. IL-10 also directly affects TNF-*α* regulation and inhibits the TNF-*α*-induced inflammatory responses [15, 21]. IL-10 -1082 SNP was associated with HBV infection susceptibility in Egyptian population [25], while IL-10 -592 SNP was associated with HBV infection clearance in Korean population [23]. A meta-analysis study showed that both IL-10 -1082A and -592A SNPs were associated with HBV clearance in Chinese population [26]. A recent study in Han population showed that A allele of SNP -592 enhanced HBV infection susceptibility, while G allele of SNP -1082 decreased HBV susceptibility, but both were not associated with HBV recovery [15]. These conflicting findings of TNF-*α* and IL-10 polymorphism effect on HBV infection warrant for more study to confirm the earlier findings.

Therefore, we determined the association of TNF-*α* (rs1800629) and IL-10 (rs1800896 and rs1800872) SNPs in the context of CHB natural history, particularly to HBeAg seroconversion, in Indonesian CHB patients.

## 2. Materials and Methods

### 2.1. Study Subjects

Subjects were recruited from Dr. Wahidin Sudirohusodo Hospital, Makassar, South Sulawesi, and from those who were referred to the Eijkman Institute, Jakarta, Indonesia, between December 2017 and December 2018. A total of 287 subjects were recruited, most were male (*n* = 205) with median age (IQR) of 44 (32–51) years. All subjects have either HBsAg positivity for more than 6 months or HBsAg-positive with negative anti-HBc IgM and have never received antiviral treatments before. All subjects were also tested negative for hepatitis C virus (HCV) and human immunodeficiency virus (HIV). Written consents were obtained from each subject following approval from the referring physicians. This study was in accordance with and approved by the Eijkman Institute Research Ethics Commission (EIREC no. 93/2015) and the Ethics Committee of Universitas Hasanuddin (1036/H4.8.4.5.31/PP36-KOMETIK/2017).

### 2.2. Serological and Biochemical Assays

All subjects were screened for HBsAg, HBeAg, and anti-HBe using Monolisa™ HBsAg ULTRA and HBeAg Ag–Ab PLUS (Bio-Rad, France) immunoassay kits according to the manufacturer's instructions. Alanine aminotransferase (ALT) and aspartate aminotransferase (AST) levels were determined using standard biochemical procedures.

### 2.3. HBV DNA Viral Load and HBV Genotyping

HBV DNA viral load was determined from 500 *μ*L of serum using quantitative real-time polymerase chain reaction (PCR) Cobas TaqMan™ HBV Test (Roche Diagnostics, USA) according to the manufacturer's instructions, with a linearity range of 10 to 10^9^ IU/mL. HBV genotype was determined by direct sequencing method of HBV S gene fragments. All sequences were aligned with HBV reference sequences and used construction of phylogenetic tree with Kimura-2 parameter, neighbour-joining algorithm, and 1,000 bootstrap resampling in MEGA ver. 5.0 (MEGA, USA) [7].

### 2.4. SNP Detection

Genomic DNA was extracted from 100 *μ*L of whole blood sample using Blood DNA Mini Kit (Geneaid Biotech, Taiwan) following the instruction manual. SNP genotyping was performed using polymerase chain reaction with restriction fragment length polymorphism (PCR-RFLP) with specific primers and restriction enzymes. PCR was performed in a 25 *μ*L reaction with 1 unit of *Taq* platinum DNA polymerase (Invitrogen, USA) and 400 nM concentration of each primers. PCR conditions were as follows: 94°C for 5 min, 35 cycles of 94°C for 30 s, 58°C or 50°C for 30 s, 72°C for 60 s, and 72°C for 5 min. RFLP digestion was carried out in a volume of 10 *μ*L with 1 unit of restriction enzyme and 5 *μ*L (0.1-0.5 *μ*g) of PCR product in NEB or CutSmart buffer (NEB, USA) for 2 h in 37°C. Two micrograms of digested products was separated in an agarose gel electrophoresis and visualized using the gel imaging systems (Bio-Rad, USA).

TNF-*α* SNP rs1800629 (-308 G>A) was detected using 5′-TGG AGG CAA TAG GTT TTG AGG GCC AT-3′ (forward primer) and 5′-TCA TCT GGA GGA AGC CGT A-3′ (reverse primer) and digested using *Nco*I restriction enzyme. IL-10 SNP rs1800872 (-592 A>C) was detected using 5′-GGT GAG CAC TAC CTG ACT AGC-3′ (forward primer) and 5′-CCT AGG TCA CAG TGA CGT GG-3′ (reverse primer) and digested using *Rsa*I restriction enzyme. IL-10 SNP rs1800896 (-1082 G>A) was detected using 5′-TCT GAA GAA GTC CTG ATG TCA CTG-3′ (forward primer) and 5′-ACT TTC ATC TTA CCT ATC CCT ACT TCC-3′ (reverse primer) and digested using *Mnl*I restriction enzyme.

### 2.5. CHB Phase Categorization

All subjects were categorized into different CHB natural history phases, immune tolerance (IT), immune clearance (IC), low/no replicative (LR), and e-negative hepatitis (ENH) [3], based on their HBeAg serostatus, HBV viral loads, and ALT levels [27]. HBeAg-positive patients were categorized into either IT or IC. Those with ALT levels less than 2x the normal level were categorized into IT, while those with ALT levels more than 2x the normal level were categorized into IC. Those with negative HBeAg, HBV viral load less than 2,000 IU/mL, and ALT levels equal to or less than 2x normal were classified as LR, while those with negative HBeAg, HBV viral load greater than 2,000 IU/mL, and ALT levels greater than 2x normal were classified as ENH.

### 2.6. Statistical Analysis

The categorical variables were analyzed using the chi-square Pearson or Fisher's exact test, while categorical and continuous variables were analyzed using one-way ANOVA or Kruskal-Wallis test. The Hardy-Weinberg equilibrium (HWE) is calculated based on an assumption that the alleles were distributed equally to the wild-type and mutants. The frequencies of the SNP genotypes among different CHB phases were compared. The genotype frequency represented the frequency of the major homozygous (MM), heterozygous (Mm), and minor homozygous (mm), which correspond to the specific allele of each SNP ID (SNP rs1800629 correspond to G/A, SNP rs1800872 correspond to A/C, and SNP rs1800896 correspond to G/A). The association between SNP genotypes with the dynamics of CHB was determined using logistic regression. The 95% confidence interval (95% CI) and the odds ratio (OR) were presented. Three different genetic association models were calculated: (1) Mendelian assumption (major homozygote (MM) vs. heterozygote (Mm), major homozygote (MM) vs. minor homozygote (mm)), (2) dominant assumption (major homozygote and heterozygote (MM+Mm) vs. minor homozygote (mm)), and (3) recessive assumption (major homozygote (MM) vs. heterozygote and minor homozygote (MM+mm)). A multivariate binomial logistic regression was used to determine factors independently associated with the CHB dynamics using backward elimination. For multivariate regression analysis, OR and 95% CI were calculated using the most abundant SNP genotype (wildtype) and female gender as references using the three genetic association models. *P* < 0.05 was considered as statistically significant. The statistical analyses were performed using SPSS ver. 25 (IBM, USA) and R ver. 3.5.3 (R Core Team, 2019).

## 3. Results

### 3.1. Subject Characteristics

The subjects' characteristics are summarized in Table 1. The CHB patients were categorized into their respective CHB phase resulting in 74 patients in IT, 28 in IC, 104 in LR, and 81 in ENH. Subjects were found gradually older from the IT to ENH phase (*P* < 0.001). Males predominate in all CHB phases (*P* = 0.422). There were significant differences for ALT, AST, and viral load levels between each CHB phase (*P* < 0.01).

Three SNPs, rs1800629 (TNF-*α*, -308 G>A), rs1800872 (IL-10, -592 A>C), and SNP rs1800896 (IL-10, -1082 G>A), were analyzed for all the subjects. For TNF-*α* -308 SNP, we identified 173 subjects with GG genotype, 5 with GA genotype, and 3 with AA genotype. The genotype distribution of this SNP met the HWE criterion (Table 2). For IL-10 -592 SNP, we identified 139 subjects with genotype AA, 89 with AC genotype, and 21 with CC genotype. Like TNF-*α* -308 SNP, the genotype distribution of IL-10 -592 SNP also met the HWE criterion. For IL-10 -1082 SNP, all the tested subjects carried the GG genotype, and none of the heterozygote and minor homozygote genotypes was identified. Therefore, IL-10 -1082 genotype distribution was highly skewed and HWE could not be calculated.

### 3.2. Associations between SNPs of TNF-*α* -308 and IL-10 -592 and CHB Dynamics

To determine the associations between TNF-*α* -308 and IL-10 -592 variants and the dynamics of CHB natural history, we compared the allele frequency of TNF-*α* -308 and IL-10 -592 in each CHB phase. The minor allele of TNF-*α* -308 (A) was observed at frequencies of 3.7%, 8.8%, 1.3%, and 3.1% in IT, IC, LR, and ENH phase, respectively. On the other hand, the minor allele of IL-10 -592 (C) was observed at frequencies of 38.6%, 33.3%, 28.4%, and 23.1% in IT, IC, LR, and ENH phase (Table 2). No difference between females and males was observed for all allele frequencies. However, since the frequencies of the TNF-*α* -308 minor alleles were too small, TNF-*α* -308 genotypes were excluded from further statistical genetic analyses.

The association of the IL-10 -592 SNP with CHB dynamics was determined using the three genetic models to look at three different events: (1) IT and IC progression to LR (HBeAg-seroconversion), (2) IT and IC progression to ENH (No HBeAg-seroconversion), and (3) LR (HBeAg-seroconversion) progression to ENH (No HBeAg-seroconversion). For our analysis, we combined both the HBeAg-positive phases, the IT and IC phases, in the logistic regression analysis since separate analysis of each phase revealed no significant association between SNP genotypes and CHB phase progression (Supplementary Table 1). The IL-10 -592 CC genotype was used as a reference genotype since frequencies of the CC genotype were low in the four CHB phases compared to the other two IL-10 -592 genotypes. This study did not find any significant association between IL-10 -592A/C polymorphism and each of the progression IT and IC to LR, IT and IC to ENH, and LR to ENH phases in all comparison gene models (AA vs. AC, AA vs. CC, AA+AC vs. CC, and AA vs. AC+CC), as shown in Table 3.

### 3.3. Associations of Multiple Host Factors and CHB Dynamics

**Figure 1:**
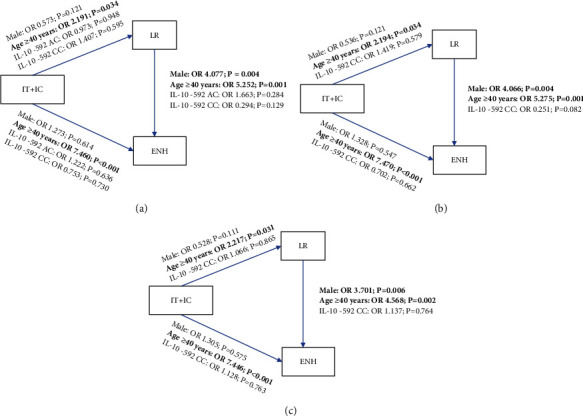
The effect of host factors on CHB phase progression as analyzed using three different genetic association models. (a) Mendelian assumption model: major homozygote vs. heterozygote and major homozygote vs. minor heterozygote. (b) Dominant assumption model: major homozygote and heterozygote vs. minor homozygote. (c) Recessive assumption model: major homozygote vs. heterozygote and minor homozygote. Host factors analyzed were male gender, age ≥ 40 years, and IL-10 -592 genotype against different CHB phase progression: IT+IC progression to LR, IT+IC progression to ENH, and LR progression to ENH. Age was significantly associated with all CHB phase progression in all three genetic association models, while male gender was only significantly associated with the LR to ENH progression (*P* < 0.05; shown in bold).

Since our bivariate logistic regression analyses did not include age and gender as confounding factors, we performed a multivariate binomial logistic regression using backward elimination to determine any host factors independently associated with the CHB dynamics. Using the Mendelian assumption, we used the abundant (wildtype) IL-10 -592 genotype and female gender as references (Table 4 and Figure 1). Subjects' age (40 years old) was significantly associated with IT and IC progression to LR (OR: 2.191, 95% CI 1.067–4.578, *P* = 0.034), IT and IC progression to ENH (OR: 7.460, 95% CI 3.316–18.310, *P* = 0.001), and LR progression to ENH (OR: 5.252, 95% CI 2.010–14.858, *P* = 0.001). Male gender was associated with LR progression to ENH (OR: 4.077, 95% CI: 1.605–11.023, *P* = 0.004). No IL-10 -592 genotypes were associated with CHB phase progression. A similar result was observed in multivariate binomial logistic regression using the dominant assumption, where genotype AC was included with the dominant genotype (AA) (Figure 1 and Supplementary Table 2). Age was found to be a significant predictor of IT and IC progression to LR (OR: 2.194, 95% CI 1.069–4.580, *P* = 0.034) and IT and IC progression to ENH (OR: 7.470, 95% CI 3.323–18.320, *P* = 0.001). On the other hand, LR progression to ENH was significantly associated with both age (OR: 5.275, 95% CI 2.013–14.954, *P* = 0.001) and male gender (OR: 4.066, 95% CI 1.597–11.005, *P* = 0.004). Similarly, a separate multivariate binomial logistic regression using the recessive assumption, where genotype AC was included with the recessive genotype (CC) (Figure 1 and Supplementary Table 3), also showed that the subject's age was associated with phase progression from IT and IC to LR (OR: 2.217, 95% CI 1.081–4.624, *P* = 0.031), IT and IC to ENH (OR: 7.446, 95% CI 3.313–18.255, *P* = 0.001), and LR to ENH (OR: 4.568, 95% CI 1.785–12.584, *P* = 0.002). The gender of the subject (male) was only associated with the progression of LR to ENH (OR: 3.701, 95% CI 1.486–9.731, *P* = 0.006). Again, no IL-10 -592 genotype was found to be significantly associated with CHB phase progression.

## 4. Discussion

Chronic HBV infection is a multifactorial disease where the disease outcome is related to both viral and host genetic factors. Among the host factors, gene polymorphisms of several immune regulators have been studied in association with CHB progression, including human leukocyte antigen (HLA) and cytokines TNF-*α*, IL-6, and IL-10 [26, 28, 29]. Despite the numerous available studies, the association between host gene polymorphism and HBV disease progression remains inconclusive, and most of these studies often contradict each other, even when they were done in similar populations [16, 24, 30]. In addition, most of these studies are also focused on CHB disease progression by comparing CHB patients with healthy or spontaneously recovered controls [30]. Here, we focused instead on the progression within the CHB natural history, specifically on the effect of the TNF-*α* and IL-10 genetic polymorphisms on HBeAg seroconversion, as one of the important milestones in CHB disease progression. In addition, looking at the correlation between genetic determinants and HBeAg seroconversion will allow us to identify specific factors that may influence the worsening disease progression in HBeAg-negative patients, whose proportion has been increasing in recent years.

TNF-*α*, as a proinflammatory cytokine, plays an important role in the clinical manifestation of various autoimmune conditions and infectious diseases [31]. Increased levels of TNF-*α* are observed in the plasma of CHB patients and have been associated with CHB-related liver disease progression [18, 31]. On the other hand, IL-10 is an immunoregulatory Th2 cytokine that may determine the balance between inflammatory and anti-inflammatory responses since it inhibits the expression of several other cytokines, including TNF-*α* [30, 32]. In addition, IL-10 level is associated with several of its gene polymorphisms and is associated with liver fibrosis progression and cirrhosis susceptibility in HBV-infected patients [16].

In this study, we looked at the effects of TNF-*α* -308 and IL-10 -592 and -1082 gene polymorphisms on CHB natural history phase progression. In contrast, the GG genotype of TNF-*α* -308 and the AA genotype of IL-10 -592 predominate in our CHB subjects, but both SNP genotype distributions were still within the HWE criterion. The IL-10 -1082 SNP deviated from the HWE criterion since only the G allele and GG genotype were identified. The GG genotype of the IL-10 -1082 SNP was associated with higher IL-10 levels [33]. It is worth noting that a study on IL-10 -1082 polymorphisms in Malaysia, which has a similar multiethnic genetic make-up to Indonesia, revealed that the A allele of IL-10 -1082 SNP, rather than the G allele, had higher frequencies in their study population [34]. The differences in this finding may be attributed to laboratory or genotyping factors, sample stratification, or evolution-random changes [35, 36].

For TNF-*α* -308, the G allele and GG genotype were found to be predominant, with only a small portion of the mixed GA and mutant AA genotype. However, the SNP genotype distribution met the HWE criterion. A recent study revealed that TNF-*α* -308 was not associated with HBV viral load or liver inflammation and fibrosis markers in a Caucasian population study [31]. However, the GG genotype of TNF-*α* -308 has been associated with higher serum TNF-*α* levels and susceptibility to HBV infection in an Asian population study, compared to the mutant AA genotype [37]. Furthermore, high levels of TNF-*α* in HBV infection have been associated with more severe liver disease symptoms [18, 38]. In contrast, a meta-analysis concluded that the A allele of TNF-*α* -308 was the one related to the increased TNF-*α* transcriptional activation and production, resulting in effective viral clearance in HBV infection [39]. Meanwhile, another meta-analysis study concluded that AA and GA genotypes of TNF-*α* -308 polymorphisms were associated with an increased risk of HBV-related HCC in both Caucasian and Asian populations [40]. The contradictory findings regarding TNF-*α* -308 alleles and HBV outcome may be due to different population genetic backgrounds or the presence of other TNF-*α* polymorphisms that may influence the regulation of TNF-*α* circulating level [39, 41]. It has been shown that there might be different regulatory processes for TNF-*α* at the circulating and local levels, in which specific TNF-*α* polymorphisms may only affect the regulation at the local level [41] Here, we could not determine the associations between TNF-*α* -308 SNPs and CHB phase progression since the minor allele A frequencies were too low to be used for further analysis.

The A allele and AA of IL-10 -592 were found predominantly with mixed GA genotypes, but all SNP genotype distributions met the HWE criterion. Furthermore, the IL-10 -592 (A>C) polymorphism has been linked to HCC in CHB patients from the Asian population [24]. Here, we determined the associations between IL-10 -592 SNPs and CHB phase progression from HBeAg-positive to HBeAg-negative phases using three different genetic association models but did not find any significant associations. This is surprising since SNP IL-10 -592 has been previously connected with HBV susceptibility and an increase in serum ALT levels in Asian HBV patients [15]. In addition, a subsequent study revealed that IL-10 -592 AC interacted with IL-4 -589 CC/CT and had a synergistic effect on liver inflammatory injury in HBV-infected patients, causing the manifestation of up to 67.64% more liver injury [42]. In addition, the IL-10 -1082 alleles have been reported to be in linkage equilibrium with IL-10 -819 and -592, resulting in three different haplotype [43], wherein two out of three haplotypes, the -1082A/-819T/-592A and the -1082A/-819C/-592C, were identified in a meta-analysis to be associated with HBV disease progression in Asians [44].

Our multivariate logistic regression analysis, which included the IL-10 -592 genotype as well as other host factors such as age and gender, revealed that, while the IL-10 -592 genotype was not associated with HBeAg seroconversion, the subject's age (40 years old) was significantly associated with progression from IT and IC (HBeAg-positive phase) to either LR or ENH (HBeAg-negative phase) and even progression from LR to ENH. In addition, the male gender was also significantly associated with the progression from LR to ENH. Age and male gender have been reported as crucial factors that play a role in CHB progression and have been correlated with the development of liver cirrhosis and cancer [45, 46]. Our results showed that subjects older than 40 years old have an increased risk of disease progression, with or without HBeAg seroconversion, and even for disease progression with the HBeAg-negative phases. Age has been identified as an important factor in HBV-related disease manifestation as age upon infection defines the chronicity of HBV infection [8] and subsequent HBeAg seroconversion in later life [5, 33, 47]. HBeAg seroconversion is also an important clinical prerequisite for the desirable HBsAg clearance, which is associated with very low disease progression and is considered the true marker for HBV cure [5]. Furthermore, since HBV is prone to mutation, the accumulation of viral mutations over time might also contribute to HBV disease progression [44, 45], in those that acquire the infection early in life. In addition, it has been shown that HBV integration and clonal hepatocyte expansion, both processes that have been implicated in the initiation of hepatocarcinogenesis, occur in the early phase of chronic HBV infection [48].

In our study, the male gender is associated with progression within the HBeAg-negative phase. This strong gender disparity has been shown previously in chronic HBV infection where males have a higher susceptibility to HBV infection [2]. Gender effects on CHB progression and liver disease manifestation have also been observed previously and are believed to be the effect of sex hormones, gender-specific behaviours, and environmental factors. The gender effect on viral infection may be caused by the difference in the interaction between sex steroids and immune effectors, testosterone on males, and estradiol on females [33]. Indeed, a recent study has shown that the androgen and estrogen receptors have the opposite effect on HBV EnhI activity and subsequent viral replication [49], confirming the proposed role of sex hormones in the immune response regulation during HBV infection and disease progression. Moreover, the genes that regulate the immune response during viral infection were localized or their expressions were controlled by other genes located on the sex chromosomes, via specific binding to hormone receptors in the immune cells [50, 51]. Androgen, which suppresses the immune response, may directly interact with the integrated HBV genome in infected hepatocytes to activate the transcription of HBV oncoprotein. Meanwhile, estrogen and estradiol protect liver cells from damage due to inflammation, apoptosis, and oxidative stress, which may contribute to further carcinogenesis development [29, 44]. These HBV dimorphic immune responses add to the complexity of CHB progression and disease manifestation.

Our study attempted to identify host determinants' effects on CHB natural history phase progression using HBeAg seroconversion as an important delineation time point. Although we managed to determine the frequencies of TNF-*α* -308, IL-10 -592, and IL-10 -1082 genotypes and alleles in our subjects, the association of TNF-*α* and IL-10 SNP genotypes and CHB phase progression was not significant. However, the multivariate logistic regression analysis showed that age and male gender have a significant association with CHB phase progression, with or without HBeAg seroconversion. A more comprehensive study involving more subjects with good subject stratification into distinct CHB phases and the addition of other TNF-*α* and IL-10 SNPs and/or other cytokine polymorphisms is required to fully understand the association of any host gene polymorphism with disease progression in CHB infection.

## 5. Conclusions

We tried to define specific host factor association with CHB phase progression. And, since CHB infection is complex, it would be beneficial to study not only the effect of host determinants but also the viral factor effects on CHB phase progression. This study is part of a bigger study on the identification of both host and viral factors that have a crucial role in disease progression in CHB patients in Indonesia. A follow-up study on specific viral factors that were important in CHB progression and HBeAg seroconversion would hopefully paint a more complete picture of the complex interaction between host, viral, and environmental factors in HBV-related liver disease progression in the hope of improving current understanding of HBV pathogenesis and disease management.

## Figures and Tables

**Table 1:** Characteristics of study populations.

Parameter	Overall (*n* = 287)	HBeAg positive		HBeAg negative		*P* value
		IT (*n* = 74)	IC (*n* = 28)	LR (*n* = 104)	ENH (*n* = 81)	
Age (years)	44 (32–51)	36 (28–48)	36 (30–51)	43 (32.3–51)	49 (41–55.5)	<0.001
<40 years (%)	112 (39.0)	39 (52.7)	16 (57.1)	42 (40.0)	15 (18.5)	<0.001
≥40 years (%)	175 (61.0)	35 (47.3)	12 (42.9)	62 (60.0)	66 (81.5)	
Gender						
Male (%)	205 (71.4)	53 (71.6)	18 (64.3)	71 (68.3)	63 (77.8)	0.422
Female (%)	82 (28.6)	21 (28.4)	10 (35.7)	33 (31.7)	18 (22.2)	
AST (U/L)	34 (24–58)	28 (22–39)	112 (81–149)	27 (20.8–33.3)	62.5 (43.3–120)	<0.001
ALT (U/L)	38 (24–69)	31 (20–46.5)	116 (85–179)	29 (19.3–42)	63 (36.5–105)	<0.001
HBV DNA (log_10_ IU/mL)	4.86 (2.47–6.59)	6.07 (3.68–8.03)	6.27 (5.18–6.97)	2.13 (1.25–2.68)	5.26 (4.27–6.52)	0.002

IT: immune tolerant; IC: immune clearance; LR: low replicative; ENH: e-negative hepatitis. Data are median (IQR) or *n* (%) or *n*/*N* (%). *P* values were calculated by ANOVA or Kruskal-Wallis test as appropriate.

**Table 2:** Distribution of TNF-*α* -308, IL-10 -592, and IL-10 -1082 genotypes and alleles in different phases of natural history of chronic hepatitis B (CHB).

Genotype	Overall	HBeAg-positive		HBeAg-negative		HWE *X*^2^	*P* value
		IT	IC	LR	ENH		
TNF-*α* -308 SNP (*n* = 181)							
GG (%)	173 (95.6)	38 (95.0)	15 (88.2)	74 (97.4)	46 (95.8)	0.298	0.370
GA (%)	5 (2.8)	1 (2.5)	1 (5.9)	2 (2.6)	1 (2.1)		
AA (%)	3 (1.7)	1 (2.5)	1 (5.9)	0	1 (2.1)		
G (%)	351 (96.9)	77 (96.3)	31 (91.2)	150 (98.7)	93 (96.9)		
A (%)	11 (3.1)	3 (3.7)	3 (8.8)	2 (1.3)	3 (3.1)		
IL-10 -592 SNP (*n* = 249)							
AA (%)	139 (55.8)	41 (61.1)	14 (51.9)	45 (51.1)	39 (58.2)	0.385	0.353
AC (%)	89 (35.7)	20 (29.9)	8 (29.6)	36 (40.9)	25 (37.3)		
CC (%)	21 (8.4)	6 (9.0)	5 (18.5)	7 (8.0)	3 (4.5)		
A (%)	367 (73.7)	102 (61.4)	36 (66.7)	126 (71.6)	103 (76.9)		
C (%)	131 (26.3)	32 (38.6)	18 (33.3)	50 (28.4)	31 (23.1)		
IL-10 -1082 SNP (*n* = 167)							
GG (%)	167 (100.0)	44 (26.3)	22 (13.2)	52 (31.1)	49 (29.3)	N/A	N/A
GA (%)	0	0	0	0	0		
AA (%)	0	0	0	0	0		
G (%)	334 (100.0)	88 (100.0)	44 (100.0)	104 (100.0)	98 (100.0)		
A (%)	0	0	0	0	0		

IT: immune tolerant; IC: immune clearance; LR: low replicative; ENH: e-negative hepatitis; HWE: Hardy-Weinberg equilibrium; *X*^2^: chi-square. Numbers (%) were presented for categorical data.

**Table 3:** The differences of proportion between SNPs of IL10-592 A/C based on natural history of chronic hepatitis B (CHB).

Dependent variables	OR	95% CI	*P* value
IT and IC to LR			
AA vs. AC	1.567	0.797–3.106	0.200
AA vs. CC	0.779	0.236–2.415	0.798
AA+AC vs. CC	0.654	0.204–1.953	0.462
AA vs. AC+CC	1.345	0.720–2.524	0.372
IT and IC to ENH			
AA vs. AC	1.257	0.604–2.617	0.604
AA vs. CC	0.388	0.065–1.600	0.240
AA+AC vs. CC	0.356	0.061–1.423	0.157
AA vs. AC+CC	1.012	0.510–2.004	1
LR to ENH			
AA vs. AC	0.803	0.389–1.643	0.612
AA vs. CC	0.498	0.078–2.367	0.503
AA+AC vs. CC	0.544	0.087–2.503	0.516
AA vs. AC+CC	0.753	0.376–1.496	0.418

IT: immune tolerant; IC: immune clearance; LR: low replicative; ENH: HBeAg-negative immune reactivation; OR: odds ratio; CI: confidence interval. ^∗∗∗^*P* < 0.001, ^∗∗^*P* < 0.01, and ^∗^*P* < 0.05.

**Table 4:** Factors independently associated with the dynamics of chronic hepatitis B (CHB) determined using multivariate binomial logistic regression with backward elimination using Mendelian assumption.

Dependent variables	OR	95% CI	*P* value
IT and IC to HBeAg-seroconversion (LR) (*n* = 133)			
Male	0.537	0.243–1.179	0.121
Age ≥ 40	2.191	1.067–4.578	0.034^∗^
IL-10 -592 AC	0.973	0.423–2.196	0.948
IL-10 -592 CC	1.407	0.389–5.067	0.595
IT and IC to no HBeAg-seroconversion (ENH) (*n* = 138)			
Male	1.273	0.500–3.320	0.614
Age ≥ 40	7.460	3.316–18.310	<0.001^∗∗∗^
IL-10 -592 AC	1.222	0.531–2.822	0.636
IL-10 -592 CC	0.753	0.134–3.679	0.730
HBeAg-seroconversion (LR) to no HBeAg-seroconversion (ENH) (*n* = 109)			
Male	4.077	1.605–11.023	0.004^∗∗^
Age ≥ 40	5.252	2.010–14.858	0.001^∗∗^
IL-10 -592 AC	1.663	0.662–4.321	0.284
IL-10 -592 CC	0.294	0.053–1.355	0.129

IT: immune tolerant; IC: immune clearance; LR: low replicative; ENH: HBeAg-negative immune reactivation; OR: odds ratio; CI: confidence interval. ^∗∗∗^*P* < 0.001, ^∗∗^*P* < 0.01, and ^∗^*P* < 0.05.

## Data Availability

The data generated and analyzed in this study were generated in a collaborative research between the Faculty of Medicine, Universitas Hasanuddin, Makassar, Indonesia; Dr. Wahidin Sudirohusodo General Hospital, Makassar, Indonesia; and the Eijkman Institute for Molecular Biology, Jakarta, Indonesia, which was part of a work of a doctoral thesis for the Degree of Doctor of Philosophy from the Universitas Hasanuddin. The original datasets used for this study are not publicly available due to the existing regulation and only can be shared upon the approval of the university, hospital, and research institute.

## Abbreviations

HBV: Hepatitis B virus
CHB: Chronic hepatitis B
TNF-*α*: Tumour necrosis factor alpha
IL-10: Interleukin-10
SNP: Single nucleotide polymorphism
IT: Immune tolerance
IC: Immune clearance
LR: Low/no replicative
ENH: e-negative hepatitis
HBeAg: Hepatitis B e antigen
anti-HBe: Antibody to HBeAg
HBsAg: Hepatitis B surface antigen
ALT: Alanine aminotransferase
AST: Aspartate aminotransferase
HWE: Hardy-Weinberg equilibrium.

## Supplementary Materials

Supplementary Materials Supplementary Table 1: demography, liver biochemistry, HBV viral load, and genetic determinants of subjects in IT and IC phases. Supplementary Table 2: factors independently associated with the dynamics of chronic hepatitis B (CHB) as determined using multivariate binomial logistic regression with backward elimination. Genotype AC was included with the dominant genotype (AA). Supplementary Table 3: factors independently associated with the dynamics of chronic hepatitis B (CHB) as determined using multivariate binomial logistic regression with backward elimination. Genotype AC was included with the recessive genotype (CC).
