# Supplementary material for: Host Factors in the Natural History of Chronic Hepatitis B: Role of Genetic Determinants
Source: Int J Hepatol. 2022 Aug 23;2022:6046677. doi: 10.1155/2022/6046677 (PMC9427277; doi:10.1155/2022/6046677)
Supplement: Supplementary Materials — Supplementary Table 1: demography, liver biochemistry, HBV viral load, and genetic determinants of subjects in IT and IC phases. Supplementary Table 2: factors independently associated with the dynamics of chronic hepatitis B (CHB) as determined using multivariate binomial logistic regression with backward elimination. Genotype AC was included with the dominant genotype (AA). Supplementary Table 3: factors independently associated with the dynamics of chronic hepatitis B (CHB) as determined using multivariate binomial logistic regression with backward elimination. Genotype AC was included with the recessive genotype (CC). [file 6046677.f1.pdf]

Supplementary Table 1. Demography, liver biochemistry, HBV viral load, and genetic determinants of subjects in IT and IC phases.

Parameter	Overall	Phase		*p*-value
		IT	IC	
Age (years), median (IQR)	36 (28–49)	36 (28–48)	36 (30–51)	0.565
Age group, n	102	74	28	
<40 years (%)	55 (53.9)	39 (52.7)	16 (57.1)	0.688
≥40 years (%)	47 (46.1)	35 (47.3)	12 (42.9)	
Gender, n	102	74	28	
Male (%)	71 (69.6)	53 (71.6)	18 (64.3)	0.472
Female (%)	31 (30.4)	21 (28.4)	10 (35.7)	
Liver biochemistry				
AST (U/l), median (IQR)	35.0 (23.0–65.5)	28.0 (22.0–39.0)	112.0 (81.0–149.0)	<0.001*
ALT (U/l), median (IQR)	39.0 (24.5–74.0)	31.0 (30.0–46.5)	116.5 (85.0–179.0)	0.002*
HBV viral load				
HBV DNA (log_{10} IU/mL)	6.25 (4.03–7.52)	6.07 (3.68–8.03)	6.27 (5.19–6.97)	0.177
Genetic determinants				
TNF-α -308 SNP, n	57	40	17	
GG, n (%)	53 (93.0)	38 (95.0)	15 (88.2)	0.658
GA, n (%)	2 (3.5)	1 (2.5)	1 (5.9)	
AA, n (%)	2 (3.5)	1 (2.5)	1 (5.9)	
IL-10 -592 SNP, n	94	67	27	
AA, n (%)	55 (58.5)	41 (61.2)	14 (51.9)	0.408
AC, n (%)	28 (29.8)	20 (29.9)	8 (29.6)	
CC, n (%)	11 (11.7)	6 (9.0)	5 (18.5)	
IL-10 -1082 SNP, n	66	44	22	
GG, n (%)	66 (100)	44 (26.3)	22 (13.2)	N/A
GA, n (%)	0	0	0	
AA, n (%)	0	0	0	

Supplementary Table 2. Factors independently associated with the dynamics of chronic hepatitis B (CHB) as determined using multivariate binomial logistic regression with backward elimination. Genotype AC was included with the dominant genotype (AA).

Dependent Variables	OR	95% CI	*p*-value
**IT and IC to HBeAg-seroconversion (LR)** (n=133)			
Male	0.536	0.243–1.178	0.121
Age ≥ 40	2.194	1.069–4.580	0.034*
IL-10 -592 CC	1.419	0.402–4.984	0.579
**IT and IC to No HBeAg-seroconversion (ENH)** (n=138)			
Male	1.328	0.531–3.410	0.547
Age ≥ 40	7.470	3.323–18.320	<0.001***
IL-10 -592 CC	0.702	0.128–3.356	0.662
**HBeAg-seroconversion (LR) to No HBeAg-seroconversion (ENH)** (n=109)			
Male	4.066	1.597–11.005	0.004**
Age ≥ 40	5.275	2.013–14.954	0.001**
IL-10 -592 CC	0.251	0.046–1.127	0.082

IT, immune tolerant; IC, immune clearance; LR, low replicative; ENH, HBeAg-negative immune reactivation; OR, odds ratio; CI, confidence interval, ***P<0.001; **P<0.01; *P<0.05.

Supplementary Table 3. Factors independently associated with the dynamics of chronic hepatitis B (CHB) as determined using multivariate binomial logistic regression with backward elimination. Genotype AC was included with the recessive genotype (CC).

Dependent Variables	OR	95% CI	*p*-value
**IT and IC to HBeAg-seroconversion (LR)** (n=133)			
Male	0.528	0.239–1.156	0.111
Age ≥ 40	2.217	1.081–4.624	0.031*
IL10 -592 CC	1.066	0.504–2.240	0.865
**IT and IC to No HBeAg-seroconversion (ENH)** (n=138)			
Male	1.305	0.517–3.385	0.575
Age ≥ 40	7.446	3.313–18.255	<0.001***
IL10 -592 CC	1.128	0.513–2.478	0.763
**HBeAg-seroconversion (LR) to No HBeAg-seroconversion (ENH)** (n=109)			
Male	3.701	1.486–9.731	0.006**
Age ≥ 40	4.568	1.785–12.584	0.002**
IL10 -592 CC	1.137	0.491–2.638	0.764

IT, immune tolerant; IC, immune clearance; LR, low replicative; ENH, HBeAg-negative immune reactivation; OR, odds ratio; CI, confidence interval, ***P<0.001; **P<0.01; *P<0.05.
